# Clinical Efficacy and Residue Depletion of 10% Enrofloxacin Enteric-Coated Granules in Pigs

**DOI:** 10.3389/fphar.2017.00294

**Published:** 2017-05-23

**Authors:** Zhixin Lei, Qianying Liu, Bing Yang, Jincheng Xiong, Kun Li, Saeed Ahmed, Liping Hong, Pin Chen, Qigai He, Jiyue Cao

**Affiliations:** ^1^Department of Veterinary Pharmacology, College of Veterinary Medicine, Huazhong Agricultural UniversityWuhan, China; ^2^National Reference Laboratory of Veterinary Drug Residues and MAO Key Laboratory for Detection of Veterinary Drug Residues, Huazhong Agriculture UniversityWuhan, China; ^3^State Key Laboratory of Agriculture Microbiology, College of Veterinary Medicine, Huazhong Agriculture UniversityWuhan, China

**Keywords:** enrofloxacin, enteric-coated granules, elimination, *APP*, *MS*, withdrawal

## Abstract

A new, more palatable formulation of 10% enrofloxacin enteric-coated granules was investigated to evaluate the pharmacokinetic effect in plasma, the residue elimination in tissues and the clinical efficacy against *Actinobacillus pleuropneumonia (APP)* and *Mycoplasam suis (MS)* in pigs. In this study, the enrofloxacin concentrations in plasma and tissues were detected using high-performance liquid chromatography with phosphate buffer (pH = 3) and acetonitrile. The pharmacokinetics and elimination of enrofloxacin enteric-coated granules were performed after oral administration at a single dose of 10 mg/kg body weight (bw) and 5 mg/kg twice per day for 5 consecutive days, respectively. The *in vivo* antibacterial efficacy and clinical effectiveness of enrofloxacin enteric-coated granules against *APP* and *MS* were assayed at 2.5, 5, 10 mg/kg, compared with tiamulin (8 mg/kg) based on establishment of *APP* and *MS* infection models. 56 *APP* strains were selected and tested for *in vitro* antibacterial activity of enrofloxacin enteric-coated granules. The main parameters of elimination half-life (t_1/2β_), T_max_, and area under the curve (AUC) were 14.99 ± 4.19, 3.99 ± 0.10, and 38.93 ± 1.52 μg h/ml, respectively, revealing that the enrofloxacin concentration remained high and with a sustainable distribution in plasma. Moreover, the analysis on the evaluation of enrofloxacin and ciprofloxacin in muscle, fat, liver and kidney showed that the recovery were more than 84% recovery in accordance with the veterinary drug residue guidelines of United States pharmacopeia, and the withdrawal periods were 4.28, 3.81, 4.84, and 3.51 days, respectively, suggesting that the withdrawal period was 5 d after oral administration of 5 mg/kg twice per day. The optimal dosage of enrofloxacin enteric-coated granules against *APP* and *MS* was 5 mg/kg, with over 90% efficacy, which was significantly different (*p* < 0.05) to the 2.5 mg/kg group, but not to the 10 mg/kg group or the positive control group (tiamulin). In conclusion, 10% enrofloxacin enteric-coated granules had significant potential for treating *APP* and *MS*, and it provided an alternative enrofloxacin palatability formulation.

## Introduction

Enrofloxacin (ENR), a classical fluoroquinolone antibiotic was developed for exclusive use in veterinary medicine for the treatment of respiratory and gastrointestinal infections. Its essential metabolite, ciprofloxacin (CP), is also widely active and effective against Gram-negative, positive aerobes, and mycoplasmas (Küng et al., 1993; Deveau, 2007; Reyesherrera et al., 2011). Although CP has not been approved for veterinary use, it is a metabolite of ENR in animals, which decreases animal mortality, promotes growth, and improves economic benefits (Sneeringer et al., 2015). With the indiscriminate use of ENR, the presence of its residues in animals may lead to adverse health effects on human, such as allergic reactions and ENR-resistant strains. In addition, sub-therapeutic metabolites of quinolones can persist in edible tissues and be ingested by human, and resistance genes may be transferred to endogenous or exogenous bacterium (Sa et al., 2008; Aliu and Sulaj, 2014; de Almeida et al., 2015; Wang et al., 2016). As a quinolone commonly used in poultry and mammalian production, ENR has a direct effect of inhibiting bacterial DNA-gyrase and topoisomerase IV enzyme activities (Ebrahimzadeh et al., 2014; Gouvêa et al., 2015). In Europe, ENR and CP are only approved for therapeutic use in animal production, and their residues could be detected in animal tissue caused by respective withdrawal periods before slaughter.

*Actinobacillus pleuropneumonia (APP)* is an obligate parasite of the porcine respiratory tract that can infect nasal cavities and tonsils of pigs (Dom et al., 1994a; Duff et al., 1996; Yoshimura et al., 2002; Jobert et al., 2010). There are 12 serotypes of biotype 1, and 6 serotypes of biotype 2, which are defined based on their surface polysaccharide antigens (Altman et al., 1990; Perry et al., 1990; Schaller et al., 2001; Bossé et al., 2002). All serotypes can be urease positive, and thus cause respiratory infection and serious economic loss. *Mycoplasam suis (MS)* is an uncultivable pathogen that can colonize the surface of porcine erythrocytes, inducing long-term hypocytosis anemia and mild fever. *MS* can also decrease reproductive efficiency in sows, and cause growth retardation in feeder pigs, causing economic reduction (Henry, 1979; Messick, 2004; Yuan et al., 2009, 2010).

Against *APP* and *MS*, ENR is widely used in veterinary clinics, but it has a bitter taste and causes irritation to the gastrointestinal tract, resulting in food refusal, vomiting, stomachache, and ventosity in animals when ENR is added to food. The application of coatings to the surface of pharmaceutical solid-dosage forms has been practiced for over 150 years. Previous reports have demonstrated enteric polymers to be safe and palatable, and have been widely accepted for use in drug products (Nykänen, 2003; Biju et al., 2004; Bushra et al., 2010). One type of enteric-coated pellet is 10% enrofloxacin enteric-coated granules (EEG), which can effectively cover the bitterness of ENR, and reduce adverse reactions in animals with sustained release. It was prepared by Shanghai Tongren Pharmaceutical Co., Ltd.

Most published studies have reported the pharmacokinetics (PK), residue and withdrawal period of ENR in broilers using HPLC, ELISA, or LC-MS/MS. The detection of ENR in edible tissues of broilers have also been evaluated in those reports (de Assis et al., 2016; Haag et al., 2016; Panzenhagen et al., 2016). Establishment of withdrawal periods are based on the depletion times of drugs, which allows the appropriate animal treatment and slaughter of treated animals, according to the requirement for the elimination and residues of drugs in animal tissues. However, there are few published reports on the residue and withdrawal period for ENR in swine.

In view of the physiological similarities of pigs with human and other animals, our study aimed to explore the metabolism, pharmacokinetic profiles, and clinical efficacy of EEG in pigs after oral administration. To ensure human food safety, this study has established compliance with withdrawal periods, maximum residue limits (MRLs), and evaluated the clinical treatment for a new formulation 10% EEG in pigs. Our results would be useful for the assessment of efficacy, safety, and effective dosage regimens and withdrawal periods of EEG for clinical use.

## Materials and methods

### Chemicals and reagents

The ENR reference standard (98.5% purity) and CP reference standard (95% purity) were purchased from Dr. Ehrenstorfer (Augsburg, Germany), 10% EEG test product, and 10% tiamulin fumarate were provided from Shanghai Tongren Pharmaceutical Co., Ltd (Shanghai, PR China). Acetonitrile and methanol were purchased from Fisher Chemicals Co., Ltd (New Jersey, USA). Phosphoric acid and triethylamine were purchased from Sinopharm Chemical Reagent Co., Ltd (Shanghai, PR China). Water was purified using the Milli-Q water purification system (Milli-Q Co., Ltd, France). All chemicals used in this study were analytical grade or higher and dissolved or diluted with deionized water.

### Bacterium

The serotype 1 of clinical *APP* pathogenic strain and *MS*, which were both isolated from Hubei Province were gifted by Huazhong Agriculture University State Key Laboratory of Microbiology. These strains were used to establish infection models for the clinical efficacy study. All strains were stored at −80°C until analysis. Prior to testing the MIC, each isolate was subcultured at least three times in tryptic soy broth (TSB) and tryptic soy agar (TSA; Qingdao Hai Bo Biological Technology Co., Ltd., Shangdong, China) containing 5% newborn calf serum (Zhejiang Tianhang Biotechnology Co., Ltd., Zhejiang, China).

### Animals

Forty-three pigs used in the study were 2-month old (20 ± 5 kg) and 120 two-and-a-half-month old healthy landrace and large white cross pigs (30 ± 5 kg) were obtained from a commercial pig farm (Hubei Jianfeng Hubei province animal husbandry Co., Ltd). The pigs were placed in separate pens, had free access to water, and were fed antibiotic-free food twice daily. The pigs were allowed a 7-day acclimation period prior to the study. Animal housing was maintained at 25 ± 2°C and 45–65% relative humidity.

All animal experiments and experimental protocols were conducted in accordance with the Guide for the Care and Use of Laboratory Animals of Huibei Provincial Laboratory Animal Public Service Center (permit number SYXK 2013-0044) and approved by the Ethics Committee of Huazhong Agricultural Univeristy, Wuhan, China.

### Susceptibility determination *in vitro*

Susceptibility determination of ENR against *APP* was performed using the agar dilution method in accordance with the CLSI recommendations in a previously described report. Strains of *APP* (2–4 μl, ~10^8^ CFU/ml) were inoculated onto TSA agar plates containing 5% newborn calf serum, with two-fold serial dilutions of ENR (0.0625–32 μg/ml). Plates of strains were incubated in the presence of CO_2_ for 48 h at 37°C. MICs were determined at the lowest drug concentrations that caused complete growth inhibition (100%). *Escherichia coli* (ATCC 25922) was used as the quality control (QC) strain to verify the results of the susceptibility testing.

### Dose administration and experiment design

#### Pharmacokinetic (PK) experiment design

Eight male pigs weighing 20 ± 5 kg were fed for 7 days before the PK experiment. All pigs received 10% EEG by oral administration at a dose of 10 mg/kg. Plasma samples (5 ml) were collected at 0.083, 0.25, 0.5, 1, 2, 3, 4, 6, 8, 12, 24, 36, 48, and 72 h after oral administering.

#### Distribution and elimination of ENR

A total of 35 male pigs weighing 25 ± 5 kg were fed for 7 days before the distribution and elimination experiment. A 5 mg/kg dose of 10% EEG was orally gavage administered twice per day for 5 consecutive days to pigs. The pigs were slaughtered through exsanguination under diethyl ether inhalation of anesthesia 1, 3, 7, 9, 11, or 13 days after ending of administration. Tissues from the liver, fat, kidney, and muscle were collected. These tissues were thoroughly rinsed with deionized water to remove residual blood, blotted to dryness, and finally homogenization was performed. Control tissues containing no drugs were also collected in order to provide control matrices.

### Sample treatment

#### Blood

Blood samples collected with anticoagulant were centrifuged for 10 min at 3,000 rpm to obtain plasma. Plasma samples were transferred to a clean sterile tube and stored at −20°C until analysis. Then, 0.5 ml plasma samples and 1 ml methanol were added to tubes, and vortexed for 2 min and centrifuged at 5,000 rpm for 10 min. The clean aqueous phase was transferred to a clean tube and dried in nitrogen at 60°C. The samples were filtered with a 0.22 μm membrane and analyzed by HPLC.

#### Tissues

Tissues (2 g) were diluted with 10 ml phosphate buffer solution into 50 ml tubes and then the mixture was vortexed and sonicated for 1 and 5 min, respectively. The obtained mixture was centrifuged at 10,000 rpm for 10 min. The supernatant was aspirated and transferred to a new centrifuge tube. The lower pellet was again processed as above. The final supernatant was obtained through activated C_18_ solid phase extraction (SPE), and then the SPE was washed with 2 ml double distilled water. When the SPE was completely dry, 1 ml of the mobile phase was extracted through SPE to obtain the final samples. Samples were injected into the HPLC for identification and quantification of the potential metabolites after filtering with a 0.22 μm membrane.

#### APP and MS infection models

For infection models, 120 pigs were randomly divided into two groups: group A for the *APP* infection model (*n* = 60) and group B for the *MS* infection model (*n* = 60). In groups A and B, 60 pigs were randomly divided into six groups, which were orally administered twice per day for 5 consecutive days 24 h after inoculation: (a) 10 mg/kg; (b) 5 mg/kg; (c) 2.5 mg/kg EEG; (d) positive group 8 mg/kg t tiamulin fumarate; (e) negative group; and (f) blank no-infection group. Each pig in the infection groups was inoculated by intranasal administration with 5 × 10^7^ to 10^8^ CFU/kg of *APP* or *MS* culture, which was previously reported to be strongly virulent. Sterile tissue (lungs) samples were collected by necropsy from dead pigs and cultured to confirm that mortality was caused by the inoculated strain.

### Clinical effectiveness

Clinical symptoms were carefully recorded for each pig, especially temperature, coughing, breathing, vomiting, prostration, and anorexia status. When evaluation indicators (temperature, coughing, etc.) were all close to or surpassed the no-infection group, the EEG efficacy groups were assessed as cured. When indicators were significantly lower compared with the positive group, the drugs were assessed as having a significant effect. When indicators were eased more than the positive group, the drug would be assessed as effective. When indicators were not alleviatived compared with the positive group, the drug would be assessed as ineffective. The presence of pathological changes in lungs after *MS* infection were scored as follows in order to make a daily clinical score: when diseased areas in lungs were 0, 1–25, 26–50, 51–75, and above 75%, they were scored as 0, 1, 2, 3, 4, respectively (Mutlu et al., 2012; Sibila et al., 2014; Cheng et al., 2017).

### HPLC condition of ENR and CP and pharmacokinetic analysis

A C^18^ reverse-phase column (250 × 4.6 mm, i.d., 5 μm, Agilent, USA) was used for HPLC, which was performed with a 278 nm detection wavelength at 30°C. The mobile phase consisted of PBS mixed with triethylamine (phase A) and acetonitrile (phase B) (v:v, 86:14).

PK parameters for plasma and tissue ENR and CP concentrations were determined using WinNonlin software (version 5.2.1, Pharsight Corporation, Mountain View, CA, USA). Drug concentrations were plotted on semi-logarithmic graphs to choose appropriate PK models.

### Statistical analysis

MIC_95_ was calculated by using SPSS software, and statistical analysis was performed with Student's *t*-test and Bonferroni revision for comparing the clinical variables of before and after treatment. The *p* < 0.05 was considered to indicate statistically significant.

## Results

### MIC distribution of *APP*

**Figure 1:**
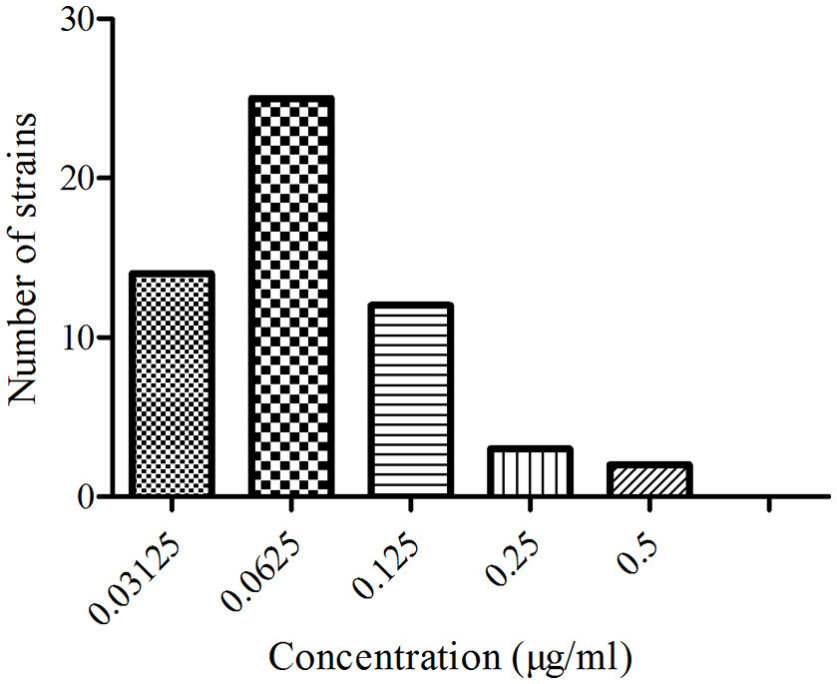
**The MIC distribution of EEG against 56 ***APP*****.

The minimal inhibitory concentration (MIC) distribution of the 56 *APP* strains to ENR are shown in Figure 1. The MIC values were 0.03125–0.5 μg/ml, and the MIC_95_ of ENR against *APP* was 0.125 μg/ml. This indicated that the *APP* strains were sensible to ENR according to the clinical and laboratory standards institute (CLSI) M100-S19 guide document. Generally MIC_95_ was the preference value if clinical extrapolations were to be made.

### Pharmacokinetic analysis of ENR in plasma by HPLC

**Figure 2:**
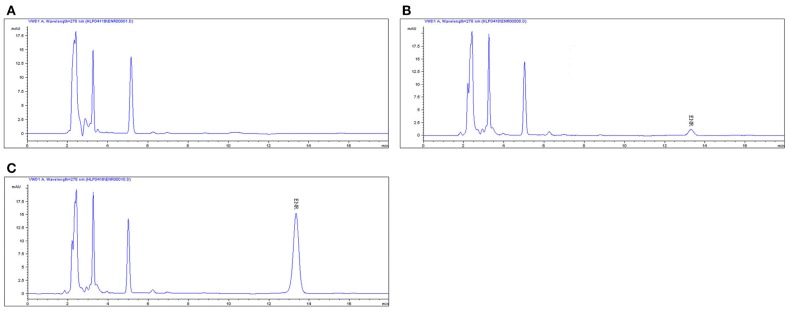
**The HPLC method for ENR quantification in plasma**. The representative HPLC chromatograms of plasma were shown: **(A)** blank plasma sample; **(B)** plasma sample at the LLOQ of 0.05 μg/ml; **(C)** plasma sample after oral administration of EEG at the point of 1 h; ENR, ENR at the peak time of 13.5 min.

The proposed method of high performance liquid chromatography (HPLC) was suitable for ENR quantification in plasma. It showed specificity and a recovery of over 82% in accordance with the veterinary drug residue guidelines of Agriculture department and United States Pharmacopeia (Gad, 2014; Millipore, 2015), and a good linear relationship from 0.05 to 10 μg/ml. The chromatogram in Figures 2A–C showed the blank Figure 2A, the lower limit of quantification (LLOQ) Figure 2B, and measured samples 1 h after oral administration in plasma Figure 2C, which indicated that the proposed method for ENR detection was specific and accurate. The typical regression equation was y = 0.008X–0.0036, *R*^2^ = 0.9999. The lower limit of determination (LLOD) was 0.025 μg/ml, and the LLOQ was 0.05 μg/ml in plasma.

**Figure 3:**
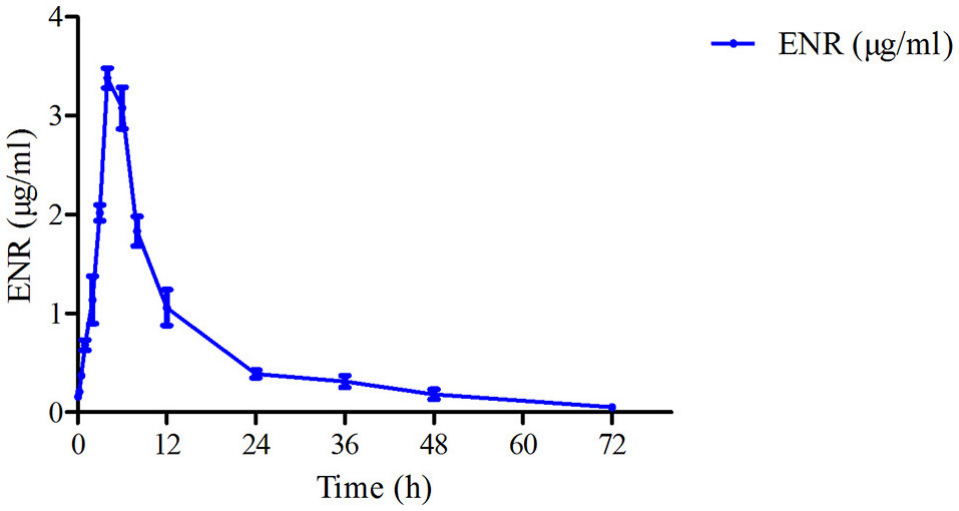
**The curve of ENR concentration–time in plasma of pigs at a dose of 10 mg/kg after oral administering**. ENR in the plasma was determined at 0.083, 0.25, 0.5, 1, 2, 3, 4, 6, 8, 12, 24, 36, 48, and 72 h.

The mean ± *SD* of ENR concentration-time profile was presented in Figure 3 after oral gavage administration, and the main PK parameters were shown in Table 1 using an absorbing two-compartment open model with a lower Akaike's Information Criterion (AIC) value (−2.43) compared other models by WinNonlin software. The results in Table 1 showed that C_max_, AUC, T_max_, and t_1/2β_, t_1/2ka_ were 3.38 ± 0.06 μg/ml, 38.93 ± 1.52 μg·h/ml, 3.99 ± 0.1, 14.99 ± 4.19, and 1.76 ± 0.29 h, respectively.

**Table 1:** **The main pharmacokinetic parameters in pigs after a single oral dose of 10% enrofloxacin enteric-coated granules (10 mg/kg.bw)**.

**Parameters**	**Units**	**(Mean ±*SD*)**
t_1/2ka_	h	1.76 ± 0.29
t_1/2α_	h	2.72 ± 0.27
t_1/2β_	h	14.99 ± 4.19
AUC	μg·h/ml	38.93 ± 1.52
C_max_	μg /ml	3.38 ± 0.06
T_max_	h	3.99 ± 0.10

*t_1/2ka_, represent absorption half-life; t_1/2α_, represent distribution half-life; t_1/2β_, represent elimination half-life; AUC, represent area under the curve; C_max_, represent peak concentration; T_max_, represent the time point to reach the peak concentration*.

### Distribution and elimination of ENR and CP in tissues

**Figure 4:**
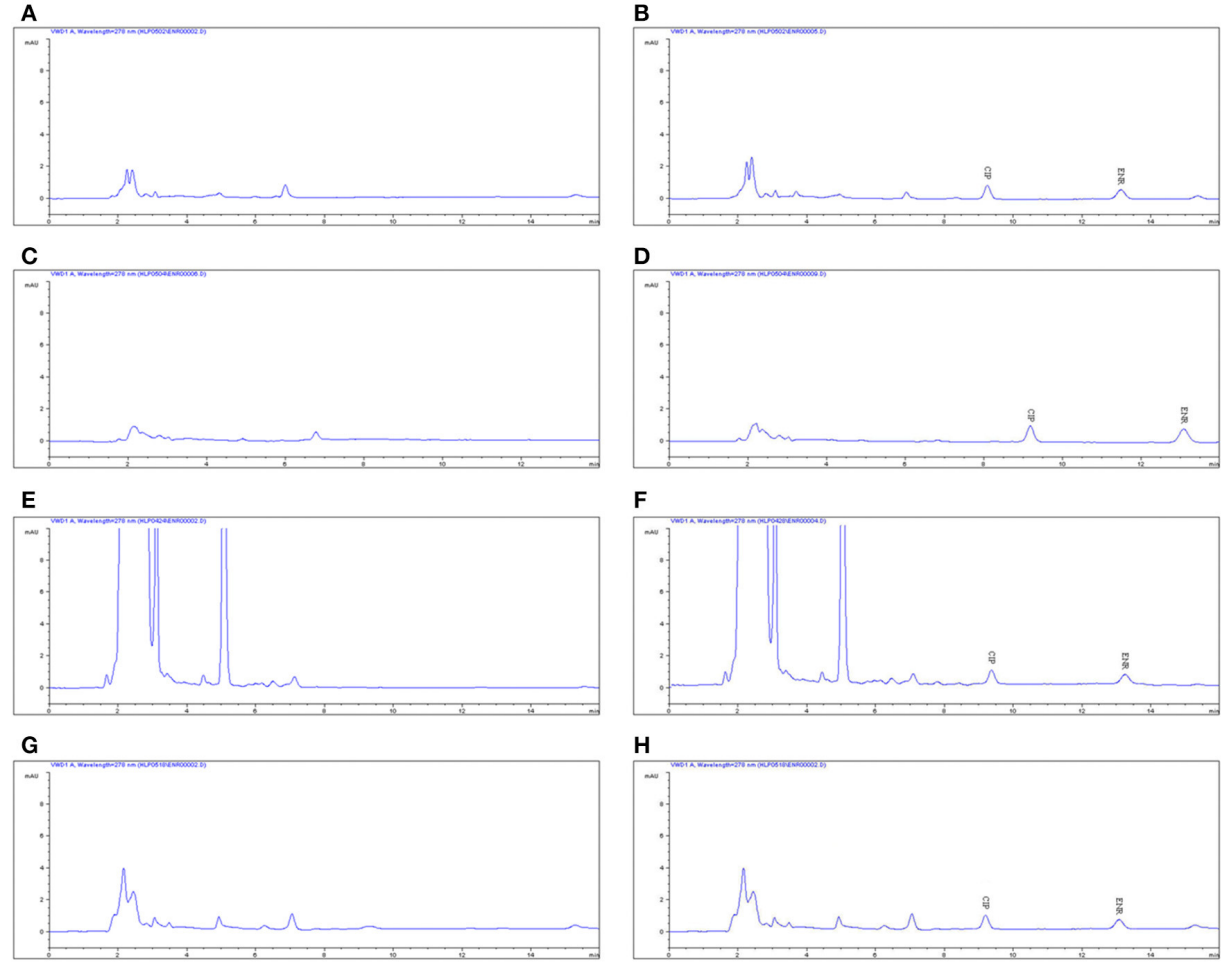
**The HPLC methods for ENR quantification in tissues**. The representative HPLC chromatograms of tissues were shown: **(A,C,E,G)** represent the blank samples in muscle, fat, liver, and kidney, respectively. **(B,D,F,H)** represent the LLOQ of 0.05 μg/ml in muscle, fat, liver, and kidney, respectively.

**Figure 5:**
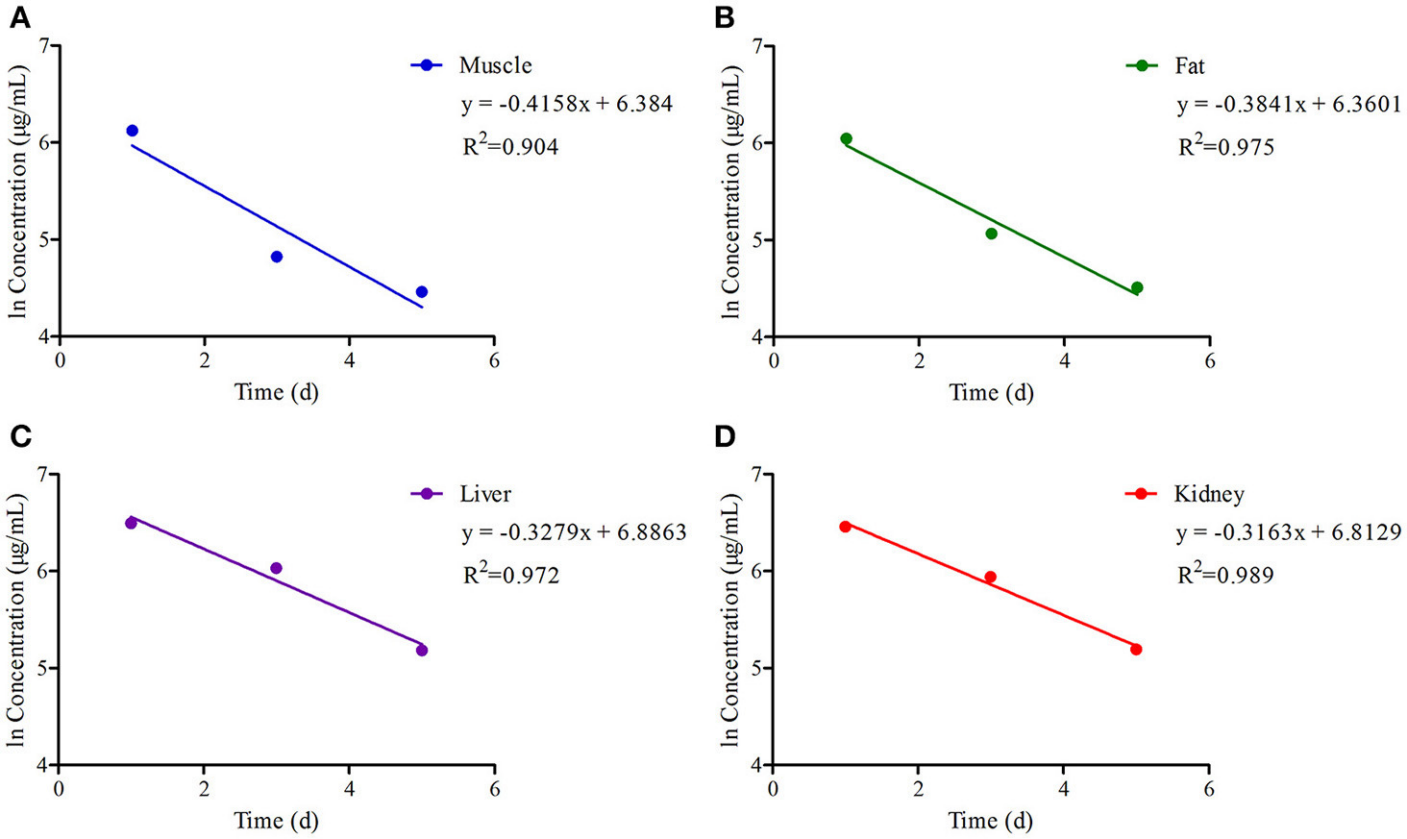
**The ln concentration-time curves of (ENR+CP) in tissues of pigs with regression lines and correlation coefficients at a dose of 5 mg/kg EEG after orally administered twice per day for 5 consecutive days**.

The chromatogram in Figures 4A–H showed the blank, LLOQs of ENR and CP in muscle, fat, liver, and kidney, respectively, which indicated the proposed methods for ENR and CP detection in tissues were specific and accurate. The lower limit of determination (LLOD) was 0.02 μg/ml, and LLOQ was 0.05 μg/ml for tissues. The coefficient of determination (*R*^2^) in 0.05–10 μg/ml of standard curves were 0.9995–0.9999 for tissues (muscle, fat, liver, and kidney). The inter-day variation was determined to be 3.6–8.0% in tissues, and the intra-day variation was 1.62–4.94%. The recovery ratios were 84.92 ± 4.72–93.83 ± 3.18% in tissues. After oral administration of 10% EEG 5 mg/kg twice per day for 5 consecutive days, distribution profiles of ENR and CP in liver and fat were similar to those in kidney and muscle, respectively (Figure 5). ENR and CP underwent a rapid and wide distribution, and reached the highest concentration in various tissues within 1 day (Figure 5). The highest concentrations were 242.4–409.4 and 188.4–270.9 μg/kg for ENR and CP, respectively, in muscle, fat, liver, kidney (Table 2). The observed drug concentrations in liver and kidney were higher than those in fat and muscle. In fat and muscle, the drug rapidly declined for the first 3 days and then decreased slowly, and was detectable after 5 days, while in liver and kidney, the drug declined rapidly for 7 days after the dose administration (Figure 5). These data in tissues represented elimination of first order kinetics, which were in accordance with the equation *C* = *C*_0_·*e*^−*kt*^, where *C* is the concentration at time t, *C*_0_ is the pre-exponential term, and *k* is the elimination rate constant. According to the equation (t_1/2*ke*_ = 0.693/*k*) t_1/2*ke*_values in each tissue were evaluated 1.67, 1.19, 2.11, 2.20 days in muscle, fat, liver, kidney, respectively (Table 3). At the same time, withdrawal periods (WDTs) were evaluated based on the provided maximum residue limit (MRL) in each tissue and the equation *MRL* = *C*_0_·*e*^−*k*(*WDT*)^, which were 4.28, 3.81, 4.84, 3.51 days in muscle, fat, liver, kidney, respectively (Table 3). As a result, the ENR and CP elimination rate of tissues from fast to slow were kidney, liver, muscle, and fat. Moreover, the WDT of tissues from short to long were kidney, fat, muscle, and liver, which were all below 5 days. Thus, the suggested WDT was 5 days after oral administration of 10% EEG 5 mg/kg twice per day.

**Table 2:** **The MRL of ENR and CIP in pig tissues at different WDT**.

**Tissues**	**Time (d)**	**ENR**	**CP**	**Total**	**MRL (μg/kg)**
Muscle	1	242.4 ± 59.4	214.6 ± 53.2	457.0	
	3	74.0 ± 11.5	50.4 ± 16.3	124.4	
	5	47.7 ± 18.6	38.9 ± 9.2	86.6	100
	7	ND	ND	ND	
	9	ND	ND	ND	
	11	ND	ND	ND	
	13	ND	ND	ND	
Fat	1	234.5 ± 53.5	188.4 ± 32.9	422.9	
	3	86.9 ± 11.6	71.6 ± 5.8	158.5	
	5	53.0 ± 11.9	38.0 ± 5.5	91.0	
	7	<LOD	ND	<LOD	100
	9	ND	ND	ND	
	11	ND	ND	ND	
	13	ND	ND	ND	
Liver	1	409.4 ± 55.4	252.1 ± 54.1	661.5	
	3	218.9 ± 29.1	197.0 ± 24.2	415.9	
	5	99.2 ± 24.3	79 ± 11.6	178.2	200
	7	ND	ND	ND	
	9	ND	ND	ND	
	11	ND	ND	ND	
	13	ND	ND	ND	
Kidney	1	367.1 ± 62.5	270.9 ± 56.1	638.0	
	3	248.0 ± 38.4	132.7 ± 26.2	380.7	
	5	69.1 ± 16.2	111.7 ± 12.2	180.8	300
	7	ND	ND	ND	
	9	ND	ND	ND	
	11	ND	ND	ND	
	13	ND	ND	ND	

*d, represent day; Total, represent the concentration of ENR + CP;ND, represent no detection*.

**Table 3:** **Summary of the elimination parameters of (ENR+CP) in tissues**.

**Tissues**	**Elimination equations**	***r***	**t_1/2**ke**_**	**WDT (d)**	**MRL (μg/kg)**
Muscle	*C* = 0.5925*e*^−0.4158**t**^	0.9509	1.67	4.28	100
Fat	*C* = 0.9181*e*^−0.5821**t**^	0.9874	1.19	3.81	100
Liver	*C* = 0.9788*e*^−0.3279**t**^	0.9861	2.11	4.84	200
Kidney	*C* = 0.9084*e*^−0.3152**t**^	0.9945	2.20	3.51	300

*r, represent correlation coefficient; t_1/2ke_, represent elimination half-life; WDT, represent withdraw period; MRL, represent the maximum residue limit*.

### Efficacy of 10% EEG in the treatment of *APP* and *MS*

There were no clinical symptoms of *APP* and *MS* in any pigs during the acclimation period prior to the infection challenge. After 24 h of the *APP* infection, the group not treated with drugs (negative group) exhibited dyspnea, cough, and fever. Swabs from the center of the lung lesions of dead pigs from the group treated with drugs (positive group) showed the satellite phenomenon around *Staphylococcus aureus* (Supplementary Figure 1) and were pink in the urease test (Supplementary Figure 2). In addition, the isolate was verified as *APP* content by PCR. These results demonstrated that the isolate from swabs was positive for *APP*, and the *APP* infection models were established successfully. After 15 days of the *MS* infection, most pigs presented rapid breathing, droopy appearance and abdominal breathing. When dead pigs from the negative group were dissected, carnification in lungs was observed (Supplementary Figure 3). The strain isolated from the lung was verified as obtaining *MS* by PCR (Supplementary Figure 4). These results demonstrated that the isolated strain from lung was positive for *MS*, and that the *MS* infection models were established successfully.

All pigs in infected groups showed lethargy and loss of appetite before treatment. The symptoms of most pigs in the treatment groups disappeared after 2 days in high-dose (10 mg/kg) and middle-dose (5 mg/kg) groups, 3 days in the positive group (tiamulin fumarate), and 4 days in the low-dose (2.5 mg/kg) group. As shown in Table 4, the ineffective ratios were 10% in high-, middle-dose, and positive groups, while the ratios in the low-dose and the negative groups were 40 and 90%, which were higher than the former groups. Moreover, the results in Table 5 showed there were no deaths in high-, middle-dose groups and positive groups, whereas the mortality rates of the low-dose groups and negative were 20 and 50%, respectively. The high-, middle-, low-dose groups displayed markedly higher effectiveness and higher average daily gain (ADG), and significantly lower detox rates than the negative group; while the high- and middle-dose groups displayed no significant difference compared with the positive group, and the low-dose group displayed lower than the positive group, in effectiveness, detox rates and ADG.

**Table 4:** **The treatment efficacy of the 10% EEG against ***APP*****.

**Teams(*n* = 10)**	**Curve**		**Significant effect**		**Effective**		**Ineffective**	
	***N***	***R* (%)**	***N***	***R* (%)**	***N***	***R* (%)**	***N***	***R* (%)**
High-dose team	6	60	2	20	1	10	1	10
Middle-dose team	5	50	3	30	1	10	1	10
Low-dose team	3	30	2	20	1	10	4	40
Positive team	5	50	3	30	1	10	1	10
Negative team	0	0	0	0	1	10	9	90
Blank	–	–	–	–	–	–	–	–

*N, represent the animal number; R, represent the ratio of N in each assessment group/the total number of each team*.

**Table 5:** **Statistical efficacy results of 10% EEG against ***APP*****.

**Teams(*n* = 10)**	**Effective rate (%)**	**Mortality rate (%)**	**Detox ratio (%)**	**ADG (g)**
High-dose team	90^a^	0^a^	20^a^	572^a^
Middle-dose team	90^a^	0^a^	30^a^	556^a^
Low-dose team	60^b^	20^b^	60^b^	354^b^
Positive team	90^a^	0^a^	30^a^	557^a^
Negative control	10	50^a^	100	239
Blank	–	–	–	710

a *Statistical significances compared with negative team are p < 0.05*,

b *Statistical significances compared with positive team are p < 0.05, ADG, represent average daily gain*.

As shown in Tables 6, 7, the ineffective ratios were 10% in high-, middle-dose, and positive groups, while the ratios in the low-dose and negative groups were 50 and 90%, which were higher than the former groups. The results in Table 6 showed there were no deaths in high-, middle-dose and positive groups, whereas the mortality rates of the negative and low-dose groups were 30 and 10%, respectively. The high-, middle-, low-dose groups displayed markedly higher effective rate and higher ADG, and significantly lower lung lesion score (LLS) than the negative group; while the high- and middle-dose groups displayed no significant difference compared with the positive group, and the low-dose group displayed lower than the positive group, in effective rate, LLS, and ADG.

**Table 6:** **The treatment efficacy of the 10% EEG against ***MS*****.

**Teams(*n* = 10)**	**Curve**		**Significant effect**		**Effective**		**Ineffective**	
	***N***	***R (%)***	***N***	***R (%)***	***N***	***R (%)***	***N***	***R (%)***
High-dose team	6	60	2	20	1	10	1	10
Middle-dose team	5	50	3	30	1	10	1	10
Low-dose team	3	30	1	10	1	10	5	50
Positive team	5	50	3	30	1	10	1	10
Negative team	0	0	0	0	1	10	9	90
Blank	–	–	–	–	–	–	–	–

*N, represent the animal number; R, represent the ratio of N in each assessment group/the total number of each team*.

**Table 7:** **Statistical efficacy results of 10% EEG against ***MS*****.

**Teams(*n* = 10)**	**Effective rate (%)**	**Mortality rate (%)**	**LLS**	**ADG (g) ADG (g)**
High-dose team	90^a^	0^a^	9^a^	832^a^
Middle-dose team	90^a^	0^a^	10^a^	821^a^
Low-dose team	50^b^	10^b^	13^b^	648^b^
Positive team	90^a^	0^a^	30^a^	822^a^
Negative control	10	30^a^	23	454
Blank	–	–	–	872

a *Statistical significances compared with negative team are p < 0.05*,

b *Statistical significances compared with positive team are p < 0.05, LLS, represent lung lesion score*.

These revealed that the efficacy of 10% EEG against *APP* and *MS* had a 90% effective ratio, which was equal to tiamulin fumarate and the dosage of 5 mg/kg twice per day for 5 consecutive days was the optimum dose.

## Discussion

The PK and residue of ENR in the serum of goats, pigs, calves, horses, sheep, and broilers have already been investigated in previous reports (Kaartinen et al., 1995; Giguère et al., 1996; Mckellar et al., 1997, 1999; Anadón et al., 1999; Elmas et al., 2001; Sang et al., 2015; Haag et al., 2016). However, most ENR products were difficult to feed in forage or water because of poor palatability. In this study, ENR coated with enteric particles was studied to evaluate PK, residue and clinical efficacy in pigs comprehensively.

For PK study in plasma, the parameters obtained in this study were compared with previous reports. C_max_, T_max_, and t_1/2β_ were 3.38 μg /ml, 3.99, 14.99 h, respectively, by oral administration of 10% EEG at a signal dose of 10 mg/kg (1 mg/kg ENR) (Table 1), while these parameter values in published studies were 0.63–1.17 μg/ml, 0.92–1.81, and 1.96–6.69 h after intramuscular administration 2.5 mg/kg of ENR (Wiuff et al., 2002, 2003; Bimazubute et al., 2010; Peng-Peng et al., 2013; Wang et al., 2016). The value of t_1/2β_ in plasma (14.99 h) in this study was double compared with previous reports (6.69 h) by Wang et al. (2016), which showed 10% EEG had a remarkably sustained release action in pigs. Moreover, C_max_ was higher than MIC_95_ for *APP* (0.125 μg/ml), and remained above 1 μg/ml for 12 h in plasma, which was higher than the MIC of most pathogenic bacteria. Thus, these results revealed that 10% EEG had high concentration, wide distribution and long-acting properties in plasma.

Regarding the accumulation and elimination kinetics of ENR and CP in pigs, total concentrations of 661.5, 638.0, 457.0, and 422.9 μg/kg were observed 1 day after drug administration in liver, kidney, muscle, and fat, respectively, and these were above MRL values for 3–4 days (Table 2). After 5 days of decontamination, the concentration of ENR and CP in each tissue were lower compared with MRL values regulated by the European Union^1^. Similar results were obtained in a previous study at a daily dose of 5 mg/kg ENR (5%) for 5 consecutive days (Garcia et al., 2005). However, in this study the administration dosage was 10 mg/kg EEG (10% ENR), which doubled that in previous work by Garcia (Garcia et al., 2005), but the ENR and CP concentrations were lower than the results by Garcia for the duration of the experiment. This result showed that 10% EEG had a faster elimination ratio in each tissue than Garcia's work. In our study, after 6 days post-administration, the drug was only detected in fat at a level below the LLOD, and not detected in any other tissues. The t_1/2*ke*_ values of kidney and liver were 2.20, 2.11 days, and these were higher than those of muscle and fat, which were 1.67, 1.19 days, respectively.

This revealed that ENR and CP were more prevalent and metabolized in kidney and liver, a result similar to published reports (Feng et al., 2005; San et al., 2007; San Martin et al., 2010; de Assis et al., 2016). This result also revealed that the liver is an appropriate target tissue for residue monitoring. For WDT evaluation of pharmacological compounds in target tissues, it was necessary to consider the administered dosage, therapy time and species. CP, as the main and crucial metabolite generated from ENR biotransformation, also had antibacterial activity for the treatment of bacterial infections (Aydemir et al., 2006; Hickerson and Carson, 2006; San et al., 2007; Schilt, 2012). According to European Medicines Agency (EMA) regulation, the target tissues for ENR and CP residue were liver, muscle and kidney^2^. However, the FDA had stated that edible tissues which eliminated slowly should also be considered as target tissue (San et al., 2007). WDTs in liver, kidney, muscle and fat were 4.84, 3.51, 4.28, and 3.81 days, respectively. Moreover, ENR was also detected after 6 days in fat, and this coincided with its slow WDT of about 3.81 days, suggesting the fat, together with liver and kidney, should also be considered as a key tissue for food safety control and toxicology concern. For the WDT of ENR, our findings in pigs were 5 days, which was similar to 5 or 6 days in poultry in previous reports (San Martin et al., 2010; Terrado-Campos et al., 2017). The previous studies revealed that after exposure to ENR at a dose of 10 mg/kg, the WDT was 5, 7, and 10 days in pigs (Delsol et al., 2004; San et al., 2007; Liu et al., 2011), and 23 days in piaractus mesopotamicus (Luo et al., 2010), most of which was far longer than that in our study (Wiuff et al., 2002; San et al., 2007; Godoy et al., 2011; Paschoal et al., 2013). Therefore, EEG had an advantage with a short WDT. And the final recommended WDT for 10% EEG was 5 days in pigs.

This study evaluated the efficacy of 10% EEG against *APP* and *MS* at high-, middle-, and low-doses compared with tiamulin fumarate. Previous reports had established a close link between the respiratory disease risk and the productivity decrease in animals (Godoy et al., 2011; Paschoal et al., 2013; Hassanpouraghdam et al., 2015; Sala et al., 2015). Generally, the strain with MIC_95_ or MIC_90_ was the preference for establishment of infection model and indicators for clinical treatment. However, correlating MIC and MIC breakpoints with clinical efficacy could not be assumed in the case of *APP* infections. To alleviate the uncertainty, we isolated the strain from the lung lesions of the dead pigs and the strain was verified to be APP, thus indicating the successful establishment of the infection model (Seah et al., 2003). A previous study had been conducted to detect the susceptibility (MIC) of 12 alternative antibiotics including ENR against 12 serotypes of 138 *APP* and acquired resistance of *APP* was found in oxytetracycline, ampicillin, and chloramphenicol while not in ENR (Reeve-Johnson, 2000). The MIC range of ENR against *APP* was from 0.06 to 0.12 μg/ml, and the MIC_90_ in the previous report was equal to MIC_95_ in this study (Dom et al., 1994b; Reeve-Johnson, 2000; Seah et al., 2003). There had been a suggestion in published study for *APP* infections in pigs that clinical demeanor and respiratory scores did not give a absolutely reliable indication of the pathology occurring in the animal (Smith et al., 1991). Although, all serotypes could cause disease, but a few reports had provided evidence that biotype 2 strains were less virulent than biotype 1 strains, and it had been reported that biotype 1 serotypes 1, 5a, 5b, 9, and 10 strains were more virulent than other biotype 1 serotypes (Smith et al., 1991; Reeve-Johnson, 1999, 2000). Thus, this study selected *APP* serotype 1 with MIC_95_ and *MS*, were selected for infection models and evaluated the *in vivo* antibacterial activity of EEG in pigs.

For evaluating the therapeutic effect, plasma concentrations should be higher or equal to the MIC (Reeve-Johnson, 1998; Cheng et al., 2017). The plasma concentrations of ENR were higher than MICs of *APP* based on the calculated pharmacokinetic values (Table 1). According to the results of MIC and pharmacokinetics in pigs, the 10, 5, 2.5 mg/kg administration dosages were set as the high-, middle-, and low-doses, respectively. For treatment efficacy of EEG against *APP* and *MS*, the efficacies of the high- and middle-dose groups were greater than that of the low-dose treatment group, while the efficacy of the middle-dose group was equal to the positive and high-dose groups, as indicators of the major efficacy parameters were not significantly different (*p* > 0.05). Therefore, the suggested therapeutic dose of 10% EEG for treatment of *APP* and *MS* was 5–10 mg/kg; the low-dose of 2.5 mg/kg could be considered as a preventive dose. In other published reports for therapeutic doses, most were i.m. administration at a higher dosage compared with that in this study (Sutter et al., 2010). Several reports gave oral administration at 5–10 mg/kg twice daily, a dosage equal to that of this study (Breitschwerdt et al., 1991; Heins et al., 2014). Previous study suggested that after treatment of ENR to serotype 1 *APP* by oral administration for 7 days, lung lesions were observed in five pigs (35.71%) with an average damage of 1.16%, and four pigs (28.57%) with 1.24% after at a dosage 40 and 150 mg/kg, respectively (Robb et al., 2007). In another study on the treatment of serotype 3 *APP* infection in the pigs, it revealed that only 150 mg/kg ENR could produce marked control of the infection in terms of reduced average severity of thoracic lesions (Dom et al., 1994b). However, the dose of 5 mg/kg of EEG produced 90% effective and 30% detox ratios in fighting *APP* infection (Tables 4, 5), and this dose was lower than those reported in the previously published studies (Herradora and Martínezgamba, 2003). Although the *MS* infection has caused more and more attention, few studies had been conducted to investigate the treatment of ENR to *MS*. Our data demonstrated an dosage of 5 mg/kg EEG produced the highest efficacy against MS, which was much lower dosage than 50 mg/kg ENR in drinking water for 3 days in one published research on the treatment of *Mycoplasma gallisepticum* (Hinz and Rottmann, 1990; Wallgren et al., 1999).

## Conclusions

In this study, PK in plasma, residue elimination in edible tissues, MICs and clinical efficacy of 10% EEG to *APP* and *MS* in pigs were assessed. The results showed that EEG had a high antibacterial activity, was fast absorbed, widely distributed, had a high concentration in plasma, and short WDT in tissues. Moreover, it presented high efficacy for *APP* and *MS*, which could provide reasonable theoretical foundation for clinical application. And EEG, as a more palatable formulation, could be used for veterinary medicine conveniently and widely.

## Author contributions

JC and QH conceived the study, JC and ZL designed the experiments. ZL, KL, and BY performed the experiments. ZL and QL wrote the manuscript. JX, LH, SA, and PC improved the language. All authors reviewed the manuscript.

### Conflict of interest statement

The authors declare that the research was conducted in the absence of any commercial or financial relationships that could be construed as a potential conflict of interest.

## Footnotes

^1^Committee for veterinary medicinal products Committee for veterinary medicinal products Enrofloxacin summary report for MRL in tissues (http://www.ema.europa.eu/docs/en_GB/document_library/Maximum_Residue_Limits_-_Report/2009/11/WC500014142.pdf.

^2^Committee for veterinary medicinal products Enrofloxacin summary report for target tissues.http://www.ema.europa.eu/docs/en_GB/document_library/Maximum_Residue_Limits_-_Report/2009/11/WC500014139.pdf
