# Supplementary figures and images for: Clinical Efficacy and Residue Depletion of 10% Enrofloxacin Enteric-Coated Granules in Pigs
Source: Front Pharmacol. 2017 May 23;8:294. doi: 10.3389/fphar.2017.00294 (PMC5440587; doi:10.3389/fphar.2017.00294)

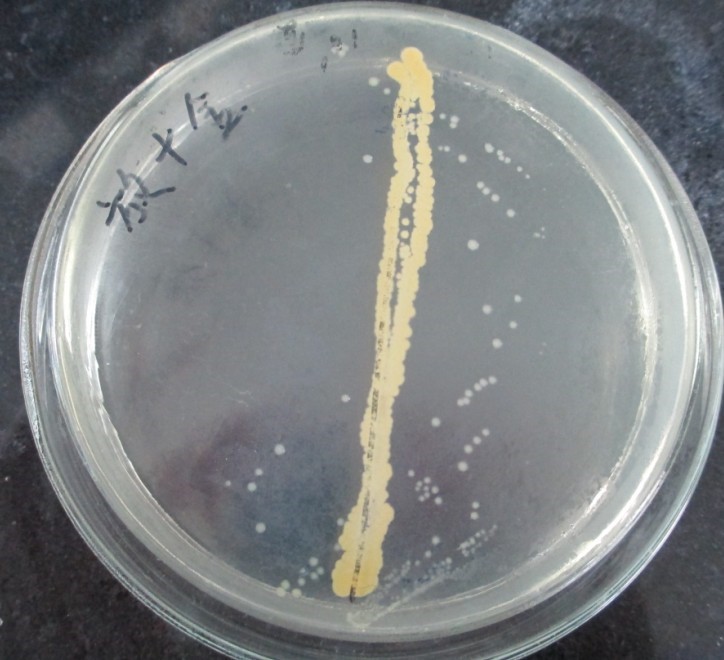

Supplement: Supplementary file 1 [file Image1.JPEG]

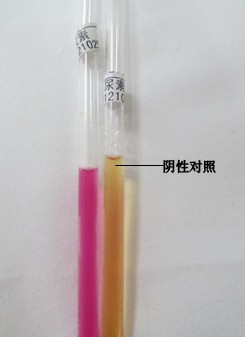

Supplement: Supplementary file 2 [file Image2.JPEG]

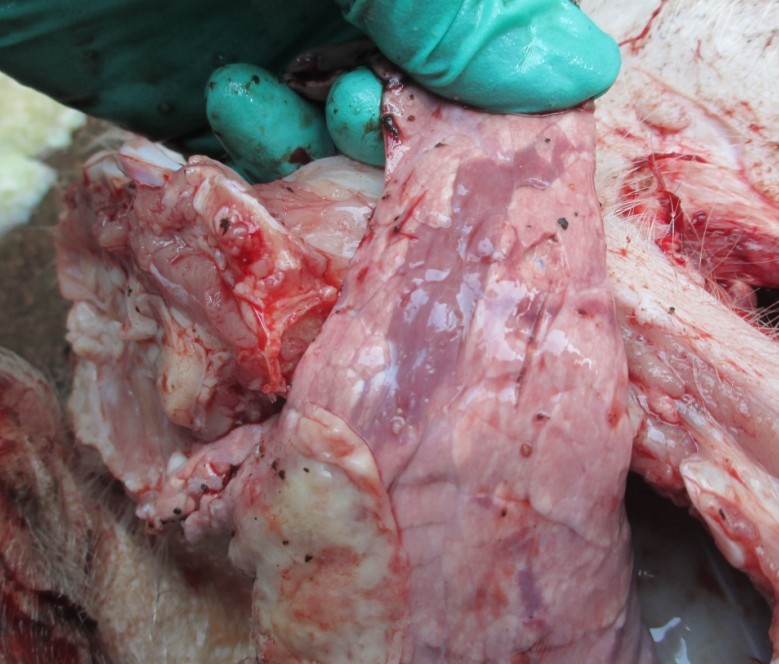

Supplement: Supplementary file 3 [file Image3.JPEG]

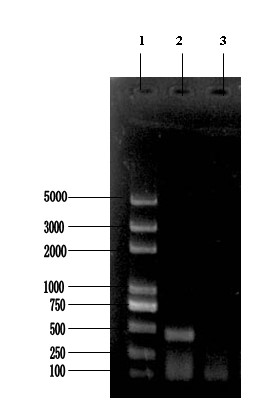

Supplement: Supplementary file 4 [file Image4.JPEG]
